# Pancreatic Beta Cell Death: Novel Potential Mechanisms in Diabetes Therapy

**DOI:** 10.1155/2018/9601801

**Published:** 2018-02-19

**Authors:** Joselyn Rojas, Valmore Bermudez, Jim Palmar, María Sofía Martínez, Luis Carlos Olivar, Manuel Nava, Daniel Tomey, Milagros Rojas, Juan Salazar, Carlos Garicano, Manuel Velasco

**Affiliations:** ^1^Pulmonary and Critical Care Medicine Department, Brigham and Women's Hospital, Harvard Medical School, Boston, MA, USA; ^2^Endocrine and Metabolic Research Center, University of Zulia, Maracaibo, Venezuela; ^3^Grupo de Investigación Altos Estudios de Frontera (ALEF), Universidad Simón Bolívar, Cúcuta, Colombia; ^4^Clinical Pharmacology Unit. School of Medicine José María Vargas, Central University of Venezuela, Caracas, Venezuela

## Abstract

**Purpose of Review:**

Describing the diverse molecular mechanisms (particularly immunological) involved in the death of the pancreatic beta cell in type 1 and type 2 diabetes mellitus.

**Recent Findings:**

Beta cell death is the final event in a series of mechanisms that, up to date, have not been entirely clarified; it represents the pathophysiological mechanism in the natural history of diabetes mellitus. These mechanisms are not limited to an apoptotic process only, which is characteristic of the immune-mediated insulitis in type 1 diabetes mellitus. They also include the action of proinflammatory cytokines, the production of reactive oxygen species, DNA fragmentation (typical of necroptosis in type 1 diabetic patients), excessive production of islet amyloid polypeptide with the consequent endoplasmic reticulum stress, disruption in autophagy mechanisms, and protein complex formation, such as the inflammasome, capable of increasing oxidative stress produced by mitochondrial damage.

**Summary:**

Necroptosis, autophagy, and pyroptosis are molecular mechanisms that modulate the survival of the pancreatic beta cell, demonstrating the importance of the immune system in glucolipotoxicity processes and the potential role for immunometabolism as another component of what once known as the “ominous octet.”

## 1. Introduction

The pancreas is a mixed gland formed by exocrine tissue represented by acinar cells that synthetize and secrete inactive digestive enzymes and by epithelial cells lining the small pancreatic ducts, which secrete great volumes of liquid rich in sodium and bicarbonate [1]. On the other hand, pancreatic endocrine tissue is represented by the islets of Langerhans constituted by alpha, gamma, and epsilon cells as well as beta cells (PBC). These constitute 70–80% of the total mass of the islet [2] and are responsible for the synthesis, storage, and secretion of insulin, a key hormone in the regulation of human metabolism [3, 4].

Insulin is a polypeptide hormone formed by 51 amino acids [5] which once bound with its receptor, mainly expressed in the liver, muscular, and adipose tissue [6], and regulates a wide number of physiological processes that comprise gene mechanisms such as cellular growth and differentiation, expression of genes that code for enzymes that trigger glycogen, and lipid and protein synthesis. Conversely, it is involved in non-gene mechanisms as well, such as regulation of key enzymes for lipid and protein metabolism and blood glucose homeostasis [7]. Given its fundamental role in glucose metabolism, any defects on insulin secretion, action or both, will lead to a cluster of metabolic alterations characterized by chronic hyperglycemia known as diabetes mellitus (DM). This can be classified according to its etiology and pathology in type 1 DM (DM1) and type 2 DM (DM2) [8].

DM1 is an autoimmune disease characterized by an absolute deficit of insulin due to selective destruction of PBC mediated by lymphocytes T and autoantibodies [8, 9]. Genetic factors have an important role in its appearance and progression [10–12]. A number of immunological events take place before the symptoms appear. Among them, the activation of self-reactive lymphocytes and their infiltration in the pancreas, followed by the release of proinflammatory cytokines such as tumor necrosis factor alpha (TNF-*α*), which united with its membrane receptor on the PBC, activate intracellular signaling pathways that end in the induction of proapoptotic mechanisms and, in some cases, cell death through necroptosis [13, 14]. In contrast with DM1, DM2 is a metabolic disease characterized by chronic hyperglycemia secondary to insulin resistance (IR). This represents a decrease in tissue response to insulin action, mainly in muscular, hepatic, and adipose tissue [5, 9]. IR is compensated through an increase in insulin secretion by PBC. However, this mechanism fails in some individuals, and it leads to failure in metabolism regulation, which causes chronic exposition of the PBC to increased glucose, free fatty acids (FFA), islet amyloid polypeptide (IAPP) levels, and cytokines such as interleukin 1-beta (IL-1*β*). This results in oxidative stress or endoplasmic reticulum (ER) stress induction in the PBC, which leads to the activation of signaling pathways of cell death such as crinophagy, autophagy, or pyroptosis, resulting in the eventual reduction of the PBC mass [15–18].

Even though PBC destruction is a key issue in the etiopathology of DM1, recent studies have demonstrated that this process is also present in other types of DM; however, cell death mechanisms of PBC differ according to type. Presently, the study of these mechanisms is considered of pivotal importance for understanding the pathogenesis of DM as well as for the development of future therapeutic targets [19].

## 2. Type 1 Diabetes Mellitus: Genetic and Environmental Factors

DM1 represents 10–15% of all DM cases [8]. This autoimmune disease is the result of a combination of genetic and environmental factors that intervene as triggers of an autoimmune reaction that selectively destroys PBC, resulting in an absolute insulin deficit [9, 20]. There are at least 20 recognized regions of the genome known as predisposing factors for DM1; however, only two of them have shown strong evidence that they are associated with it. These are human leukocyte antigen (HLA) region and the insulin gene region [21].

The HLA region is located on a 3 Mpb stretch within chromosome 6p21, in a locus that contains more than 200 genes that comprise approximately 4000 base pairs [22], grouped in three regions or classes: class I (haplotypes HLA-A, HLA-B, HLA-C, among others) [23]; its phenotypical expression is the major histocompatibility complex class I (MHC-I) expressed on the surface of all nucleated cells as well as a series of proteins involved in the regulation of natural killer cells (NK) and lymphocytes T [24]. The HLA class III region is formed by genes that code components of the complement pathway (C2, properdin, factor B, C4A, and C4B) and products with inflammatory activity such as TNF-*α* and acute phase proteins [25]. Finally, HLA region class II is conformed by haplotypes HLA-DR, HLA-DQ, and HLA-DP [23] that code the major histocompatibility complex class II (MHC-II), expressed only in antigen-presenting cells (APC). In this locus, there are also genes that code for several proteins (TAP, LMP-2, LMP-7, and HLA-DM) with antigenic processing activity [26]. HLA genes are characterized for being very polymorphic and with great variability, which is why no specific mutation is known as the direct cause of DM1 at this time. However, haplotypes associated with a greater susceptibility to this pathology have been identified [27].

Around 40% of the genetic risk associated to DM1 is related to HLA region class II, especially HLA-DR and HLA-DQ, where the haplotypes with the greatest association are DRB1∗0401 or ∗0405 and DQB1∗0301 (DR4-DQ8) [28]. Unlike region MHC class II, less than 20% of the cases are associated with mutations in the MHC class I region, in which haplotypes HLAB∗3906 or HLA-A∗2402 set susceptibility towards DM1 [29, 30]. However, mutations at the HLA locus do not explain the entire association to DM1; a lesser genetic predisposition is due to mutations outside of the HLA locus, such as the insulin gene (11p15.5). This gene is considered a susceptible locus due to a region of variable number of tandem repetitions (VNTR) that modulates insulin expression in the thymus during lymphocyte T maturation [2, 10].

However, these genetic factors cannot explain the etiology of this disease by themselves. Epidemiologic studies suggest that environmental factors play a pivotal role in the development of DM1 acting as triggers of the disease [31]. Among these factors, there are cesarean sections [32], early exposure to cow milk protein [33], vitamin D deficiency, viral infections, limited exposure to microorganisms during childhood, and childhood obesity [34]. These have all been associated with the development of DM, converging in the loss of immunologic tolerance and the participation of self-reactive T cells in susceptible patients, which later conducts to an immunological phenomenon known as “insulitis” [27].

## 3. Insulitis: The Seal of Autoimmunity

A key step in the generation of immune tolerance is antigen presentation by the APC to lymphocytes T and B. In a functional immune system, the capacity to distinguish between what is self and what is not self is fundamental. However, this capability for recognition is lost when central tolerance mechanisms fail (induced in the developing sites of lymphocytes T and B, thymus, and bone marrow, resp.), which leads to the development and expansion of self-reactive effector cells [35]. In DM1, progressive infiltration of self-reactive immune cells belonging to the innate and the adaptive immune system into the islets of Langerhans constitutes a phenomenon known as “insulitis” that represents the seal of autoimmunity [36].

Initial infiltration of the islets requires autoantigen recognition by the dendritic cells (DCs) and macrophages, which are professional APC. These autoantigens include insulin [37, 38], glutamate decarboxylase (GAD) [39], protein tyrosine phosphatase [40], insulinoma-associated antigen- (IA-) 2, and IA-2b [41], among others. These autoantigens (some of them cryptoantigens) are exposed to APCs as a result of PBC death through physiological mechanisms or as a product of viral infections that lead to their destruction [10, 42, 43].

Once the APCs process the autoantigens, the DCs migrate towards the pancreatic lymph nodes where they promote the activation of adaptive immunity through CD4 T lymphocyte activation through IL-12 release. The DCs also promote activation of lymphocytes B to plasmatic cells, which allows for antibody release against autoantigens of the PBC [31, 44–46]. T cells differentiated to Th1 CD4^+^ secrete IL-2 and interferon-gamma (IFN-*γ*), which further stimulates DC and macrophage secretion of other cytokines such as IL-1*β* and TNF-*α* that in turn promotes cytotoxic T CD8 cell migration. This leads to a progressively activated macrophage and T cell accumulation around and inside the islets [45, 47]. Additionally, inflammatory cytokines induce overexpression of MHC type I molecules in the PBC and MHC-II in the APC, increasing autoantigen presentation, which raises the susceptibility of PBC to attacks from self-reactive T lymphocytes [26]. Once activated, these self-reactive cells unleash an immune response through the release of proinflammatory cytokines that will bound with receptors located on the PBC surface and that will activate intracellular signals that will end in the death of the cell [48].

## 4. DM1: Death of the Pancreatic Beta Cell

As it has been previously discussed, DM1 is an autoimmune disease of chronic evolution in which the process responsible for PBC death progresses through the years. Clinical symptoms of DM1 appear when more than 70% of the PBC mass is destroyed [49]. PBC destruction mechanisms in DM1 have not been completely clarified; however, a great importance has been attributed to the following mechanisms [13]: (1) expression of the apoptosis stimulating fragment (Fas) and its ligand Fas-L on the surface of CD8^+^ T-activated cells and PBC, respectively; (2) secretion of proinflammatory cytokines such as IL-1*β*, TNF-*α*, and IFN-*γ* by the different immune cells infiltrated in the islets of Langerhans; (3) production of reactive oxygen species (ROS) such as nitric oxide (NO) by macrophages, DCs, and PBC. All of these mechanisms converge in the induction of a type of programmed cell death known as apoptosis. This is the main form of death observed through biopsies of islets of rats and humans with DM1 [14, 50–52].

## 5. Apoptosis and DM1: Cytokines and the Extrinsic Pathway

Kerr et al. described the term apoptosis for the first time in 1972 [53]. It is used to describe a specific morphologic pattern of cellular death that includes the following: alterations in the nuclear morphology where a condensation and fragmentation of chromatin is observed, cell shrinkage followed by plasmatic membrane blebbing, and the separation of cell fragments. These structures are known as apoptotic bodies, pack intact organelles, cytoplasm, and nuclear remains during a process called “budding” [54, 55]. *In vivo*, these apoptotic bodies are removed by phagocytic cells residing in the tissue without activating any inflammatory reaction on it. These morphologic alterations are the result of the activation of intracellular signaling pathways that involve a series of molecular events and biochemical reactions triggered by a variety of stimuli and conditions which can be physiological or pathological [56].

There are two main apoptotic signaling pathways, both involved in the death of the PBC during the development of DM [57]; the intrinsic pathway, also known as the mitochondrial pathway, is activated by different types of cell stress such as hypoxia or oxidative stress, in which proteins of the Bcl-2 family intervene [58, 59]. These are grouped into three classes: the first one inhibits apoptosis and it includes Bcl-2, Bcl-x, and MCL-1; the second class promotes apoptosis and it includes Bax and Bak, and lastly, BH3 proteins such as Bad and Bix, which favor the apoptotic signal through inhibition of antiapoptotic proteins that act as cell stress sensors, comprise the third class. Conversely, the extrinsic pathway or the pathway mediated by cell death receptors initiates with the union of ligand belonging to the TNF superfamily such as TNF-*α*, Fas-L, and its respective receptors expressed on the surface of the PBC that translates the apoptotic signal downstream through its cell death domain (DD) to other associated proteins [14, 60].

**Figure 1:**
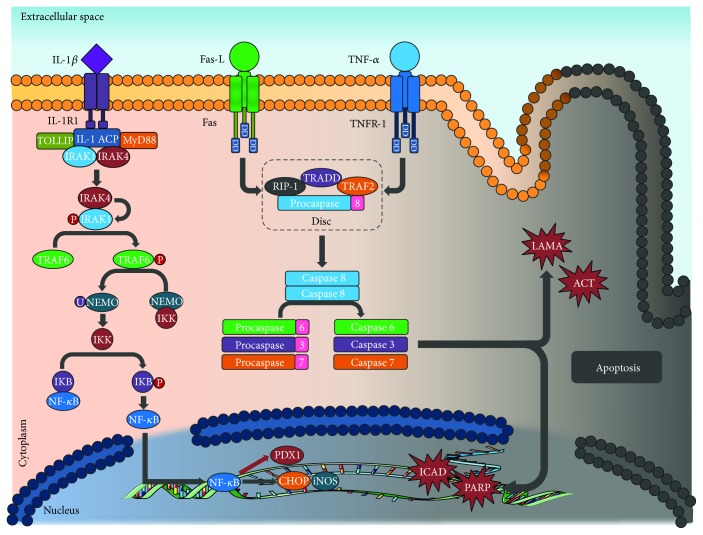
Apoptosis: the union of ligand IL1-1*β*, Fas-L, and TNF-*α* triggers a series of intracelular signals that end with caspase activation, which is capable of mediating the rupture of other proteins in aspartate residues through its cysteine residue (see text). IL-1*β*: interleukin 1 beta; Fas-L: Fas ligand; TNF-*α*: tumor necrosis factor alpha; IL-1R1: interleukin 1 receptor type 1; TNFR-1: tumor necrosis factor receptor 1; DD: death domain; TOLLIP: Toll-interacting protein; IL-1 ACP: interleukin 1 receptor accessory protein; MyD88: myeloid differentiation protein; IRAK1: interleukin 1 receptor-associated kinase 1; IRAK4: interleukin 1 receptor-associated kinase 4; TRAF6: TNF receptor-associated factor 6; NEMO: nuclear factor kappa B essential modulator; IKK: inhibitor of nuclear factor kappa B kinase; IKB: NF-*κ*B inhibitor; NF-*κ*B: nuclear factor kappa B; PDX1: insulin promoter factor 1; CHOP: CCAAT enhancer-binding protein homologous protein; iNOS: inducible nitric oxide syntase; ICAD: inhibitor of caspase-activated DNase; PARP: poly ADP-ribose polymerase; RIP1: receptor-interacting protein kinase 1; TRADD: tumor necrosis factor receptor type 1-associated death domain protein; TRAF2: TNF receptor-associated factor 2; DISC: death induction signaling complex; LAMA: laminin A; ACT: actin.

In any of the two cases, once the death signal is recognized, recruitment of the proteins in charge with executing said signal takes place. These include cysteine-aspartic proteases (caspases) [61], which are a family of proteins synthetized in their inactive form as zymogens or procaspases, each with an N-terminal domain that needs to be eliminated for their activation. Once active, they have the ability to mediate the rupture of other proteins in aspartate residues through its cysteine residue. Caspases work in a coordinated and sequential manner [62] in a process that leads to apoptosis. They are classified according to their activity: there are initiator caspases (caspases 1, 8, 9, and 10), which activate other caspases, and executioner caspases (e.g., caspases 2, 3, 6, and 7), which degrade proteins vital to the cell and are responsible for the morphologic changes suffered by the PBC [13, 54] (Figure 1).

### 5.1. Fas/Fas-L

Fas (also known as APO-1 or CD95) is a type II transmembrane protein with extracellular domains rich in cysteine and a highly conserved cytoplasmic DD, which is common in all members of the TNF receptor family [63]. Fas is expressed on the surface of several cellular types, and its interaction with its Fas-L ligand, a type II protein of 40 kDa that it is also one of the main effector molecules of CD8^+^ T lymphocytes and NK, initiates a sequence of intracellular events that lead to an apoptotic cell death through the extrinsic pathway [64].

Fas and Fas-L have both been detected on the surface of the PBC and in a high percentage of infiltrated T cells in the pancreatic islets, associating them with autoimmune destruction of the PBC during DM1 [13, 14]. Similarly, PBC normally produce Fas-L but they normally do not produce Fas at detectable levels. However, conditions associated to cell stress such as chronic exposure to elevated glucose concentrations or other cytokines such as IL-1*β* could induce Fas expression and subsequent apoptosis of the PBC [65].

Immediately after Fas-L and Fas interact, the apoptotic signal is transmitted through Fas DD to the adaptive protein associated to death domains (FADD) once the Fas receptor trimerises [66]. FADD permits the recruitment of procaspase 8, forming a signaling complex known as death induction signaling complex (DISC). Its function is the processing of procaspase 8 to its active form (caspase 8), which leads to the activation of executioner caspases downstream of the signaling pathway, like caspase 3, executing the apoptotic signal and PBC death [12, 35, 50].

### 5.2. Il-1*β*

IL-1*β* is a 17 kDa protein mainly synthetized by activated macrophages and other cellular types like monocytes, fibroblasts, and DCs as a proprotein or zymogen (pro-Il-1*β*) with no biological function that accumulates in the cytosol of the cell and that requires elimination of a protein segment for its activation [67]. Conversion of pro-IL-1*β* to its biologically active form (IL-1*β*) requires assembling of a multiprotein complex known as inflammasome and of caspase 1 activity, which splits the amino acid sequence [68]. Once activated, IL-1*β* plays a fundamental role in the amplification and maintenance of the inflammatory response through the expression of other inflammation mediators like Fas-L, as well as NO production. Therefore, this cytokine is involved in the pathogenesis of numerous metabolic ailments such as DM1, and it is capable of causing dysfunction and PBC death [13].

IL-1*β* signal is transmitted through two receptors, both expressed on the surface of the PBC. Low affinity IL-1R1 is in charge of the signal transduction to the inside of the cell, whilst IL-1R2 is a high affinity decoy receptor [67]. After the interaction of IL-1*β* with IL-1R1, conformational changes take place on the receptor that permit the coupling of the accessory protein of the IL receptor (IL-1 ACP) and the later formation of a multiprotein complex that involves the Toll-interacting protein (Toll_a_) as well as the myeloid differentiation primary response gene 88 (Myd88). This leads to the recruitment of two members of the serin/treonin kinases associated to IL-R1 (IRAK) types 1 and 4 [50], which were forming a complex in the cytosol with the Toll_a_ protein before the receptor activation. In this context, IRAK4 phosphorylates IRAK1 activating it, which permits its interaction with the TNF receptor-associated factor 6 (TRAF6) in the cytoplasm causing its activation after its phosphorylation [14].

TRAF6 is a type 3 enzyme ubiquitin ligase that mediates the activation of the inhibitor of the nuclear factor kappa-B kinase (IKK) through the ubiquitination and later degradation of the nuclear factor-kappa B essential modulator (NEMO) [69]. Once active, IKK phosphorylates the NF-*κ*B inhibitor (I*κ*B), releasing the inhibition of NF-*κ*B and allowing for its translocation from the cytoplasm to the nucleus of the PBC. [70] NF-*κ*B is a dimer that results from the combination of a family of structurally related DNA-binding proteins: p65 (RelA), RelB, c-Rel-Rel, p50, and p52 that act as transcription factors. The general term NF-kB traditionally refers to the heterodimer p50/p65 (p50/RelA), which is an apoptosis-regulating gene [71, 72]. Once inside the nucleus, NF-*κ*B induces expression of inducible nitric oxide synthase (iNOS) in the PBC. This enzyme acts as a catalyst for NO generation from L-arginine, which is capable of reacting with prosthetic groups present in transcription factors and DNA fragmentation, as well as inhibiting enzymatic activity thus decreasing glucose oxidation, oxygen consumption, ATP synthesis activity, and therefore, insulin synthesis [73]. NO may directly induce cytochrome c release from the mitochondrial membrane through the formation of peroxynitrite when combined with ROS [74]. The release of cytochrome c leads to the execution of the apoptotic signal through executioner caspase activation downstream of the signaling pathway. This is a point of convergence between the extrinsic and the intrinsic apoptosis pathways [75].

In addition to iNOS expression, NF-*κ*B might cause downregulation of transcription factors necessary for differentiation and maintenance of the PBC function, such as insulin promoter factor 1 (PDX1) [73]. It also promotes the expression of proapoptotic genes like the CHOP, GADD1, or DDIT3, related to ER stress [76].

### 5.3. TNF-*α*

TNF-*α* is a 17 kDa with 175 amino acids [77], mainly synthetized by activated macrophages, lymphocytes Y and NK cells as pro-TNF-*α* (26 kDa) anchored to the membrane. Therefore, it requires the action of the TNF-*α*-converting enzyme (TACE) for the elimination of its prodomain, consequent release in the pancreatic islet, and later union with its receptor, expressed by the PBC [78].

The TNF-*α* receptor called TNFR-1 is expressed in a ubiquitous manner in all type of cells and, unlike the TNR-2 receptor (expressed exclusively in immunological cells), it possesses a DD and therefore, it is capable of inducting apoptotic cell death [79]. After the union with the TNF-*α*, TNFR-1 trimerises and the silencer of DD proteins (SODD), which was united to the receptor, is released. This allows for the recruitment of the TNFR-1-associated death domain protein (TRADD) through its DD, which acts as an adapter protein for the assembling of other proteins such as the receptor-interacting protein kinase 1 (RIP1), TNF receptor-associated factor 2 (TRAF2), and FADD [80]. At the same time, these proteins recruit key molecules that are responsible for mediating the intracellular signal, among these, procaspase 8 and subsequent formation of the DISC complex [13–15, 35].

The DISC facilitates the autoproteolytic rupture of procaspase 8, which is responsible for its enzymatic activity and its release that leads to the activation of downstream caspases in the signaling pathway (caspases 3, 6, and 7). These will act in the proteolysis of intracellular substrates such as laminin A, poly ADP-ribose polymerase (PARP), the inhibitor of the caspase-activated DNase (ICAD), and later DNA-fragmentation, cytoskeleton protein degradation, and cellular membrane collapse. This pathway continues with the formation of apoptotic bodies and the death of the PBC [61]. However, studies have shown that TNFR-1 activation not only conducts to apoptotic cell death but also is capable of inducting another type of cell death known as necroptosis [81].

## 6. Necroptosis in DM1

Once TNF-*α* binds to its receptor, it has the capability of forming two opposing signaling complexes: DISC or IIa, which induces apoptosis, the previously described mechanism. Similarly, it can form the IIb complex [82] that appears under conditions insufficient for apoptosis initiation and that it is capable of starting a signaling pathway of programmed cell death known as necroptosis or programmed necrosis, a term introduced in the year 2003 by Chan et al. [83].

Unlike apoptosis, where caspases are the catalyst proteins for the PBC death, in necroptosis, the responsible for the downstream death signal execution is the IIb complex or necrosome. The formation of this complex is highly regulated by mutual ubiquitination and phosphorylation of proteins RIP1 and RIP3, which are part of this complex, as well as FADD, TRADD, and procaspase 8 [84]. Once activated, the necrosome unleashes diverse molecular mechanisms initiated by TNF-*α* that contribute to necroptosis execution, among which excessive ROS production and DNA fragmentation stand out [85].

Excessive ROS production mediated by the necrosome occurs through the following mechanisms: (a) RIP3 can allosterically activate glutamate-ammonia ligase or glutamine synthetase (GLUL) and glutamate dehydrogenase (GLUD1) enzymes. Both intervene in glutaminolysis, a process in which *α*-cetoglutarate is generated. As a substrate of the Krebs cycle, it increases the metabolite influx to said cycle with the consequent increase of reduction-oxidation reactions in the respiratory chain and the accumulation of ROS levels that are potentially harmful for the PBC [86]; (b) TNF-*α* also stimulates ROS production through ferritin degradation mediated by the c-Jun NH2-terminal kinase (JNK). Therefore, it increases the labile iron pool which favors ROS production through Fenton reaction, with the production of great quantities of hydroxyl radical (−OH) that participates in the peroxidation of cell membranes [87].

During this entire process, the increase of ROS concentrations initiates a vicious cycle of cell damage aggravating mitochondrial uncoupling [88]. Furthermore, lipid peroxidation favors the opening of the mitochondrial pore, allowing for the translocation of the apoptosis-inducing factor (AIF) protein to the nucleus, where it forms a complex with H2AX histone and cyclophilin A (CypA). This induces DNA fragmentation on a large scale, independently of caspases' presence [89].

**Figure 2:**
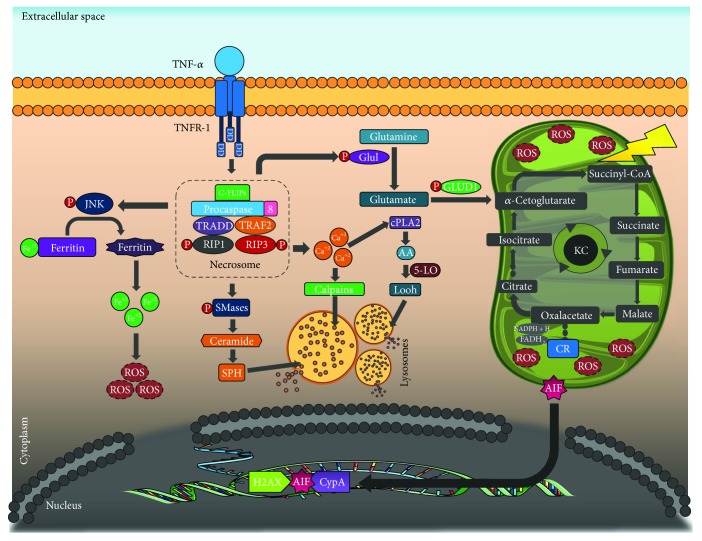
Necroptosis: after the union of TNF-*α* to its receptor, the IIb complex or necrosome can be formed. This complex induces ROS production and DNA fragmentation (see text). TNF-*α*: tumor necrosis factor alpha; TNFR-1: tumor necrosis factor receptor 1; DD: death domain; C-FLIPs: cellular FLICE inhibitory protein; JNK: c-Jun N-terminal kinase; TRADD: tumor necrosis factor receptor type 1-associated death domain protein; TRAF2: TNF receptor-associated factor 2; RIP1: receptor-interacting protein kinase 1; RIP1: receptor-interacting protein kinase 3; ROS: reactive oxygen species; SMases: sphingomyelinase; SPH: sphingosine; GLUL: glutamate-ammonia ligase or glutamine synthetase; GLUD1: glutamate dehydrogenase; cPLA2: calcium-dependent phospholipase A2; AA: arachidonic acid; 5-LO: 5-lipoxygenase; LOOH: lypid hydroperoxides; NADP: nicotinamide adenine dinucleotide phosphate; FAD: flavin adenine dinucleotide; AIF: apoptosis-inducing factor; H2AX: histone H2A variant; CypA: cyclophilin A.

Necrosome activation also favors ceramide production through the induction of the ceramidase enzyme, and it promotes cytosolic calcium increase. This activates calpains and A2 cytosolic phospholipase, capable of starting lipid peroxidation through the mobilization of arachidonic acid as substrate for the lipoxygenase enzyme. Similarly, sphingosine (SPH), calpain, and hydroperoxidases induce permeation of the lysosomal membrane, causing the migration of hydrolytic enzymes, capable of degrading vital proteins for the PBC towards the cytosol [82, 89] (Figure 2).

## 7. Type 2 Diabetes Mellitus: Insulin Resistance Mechanisms

DM2 constitutes 90–95% of the DM cases, and it is considered as an ensemble of metabolic alterations. It is characterized by chronic hyperglycemia due to the progressive loss of insulin secretion associated to the presence of IR, representing an existing defect of its activity [8]. In this sense, IR is a metabolic state in which the activity of insulin on the peripheral tissue is reduced (mainly in the muscular, adipose, and hepatic tissues), constituting the main pathophysiological display of several diseases such as obesity, metabolic syndrome (MS), polycystic ovarian syndrome (PCOS), among others [90]. Resistance to the action of these hormones is countered by the increase of its secretion by the PBC [5]. Despite the extensive scientific development that includes high precision techniques such as wide genome scanning and expression essays (microarrays), all the currently proposed mechanisms only explain a part of the phenomenon or are only applicable for a specific diabetic phenotype [9].

In addition to pathogenic mechanisms at the receptor level and on its signaling pathways, new IR mechanisms involving branch-chained amino acids have been described. These have been related to mitochondrial dysfunction, activation of the mTORC1 pathway, and regulation of the transendothelial flux of fatty acids [91, 92].

## 8. Glucotoxicity

Metabolic state influences adult *β* cell destination. At baseline, the PBC secrete insulin in response to glycaemia levels. If there is insufficient insulin amount to respond to the metabolic demand, the PBC starts to prime in order to proliferate and alleviate the stress levels [93]. In this context, due to the high blood glucose levels, the cells start to suffer changes induced by glucotoxicity, a mechanism described as irreversible function alteration of the PBC and the expression of genes as a result of the prolonged exposition to supraphysiologic glucose concentrations *in vitro* as well as *in vivo* [94].

Glucotoxicity is capable of causing dysfunction of the PBC mainly through oxidative stress. This is because the overproduction of free radicals by the electron transportation mitochondrial chain reduces the activity of the glycolytic enzyme glyceraldehyde-3-phosphate dehydrogenase (GAPDH) [95]. Therefore, when its activity is inhibited, there is an increase in concentrations of all the intermediate glycolytic compounds upstream of the enzymatic reaction catalyzed by GAPDH, for example, the rise in glyceraldehyde-3-phosphate concentration or in the levels of fructose-6-phosphate [96]. Finally, inhibition of GAPDH increases the intracellular levels of the first metabolite of the glycolytic pathway (glucose). Consequently, substrate for the polyol pathway increases, and the aldose reductase enzyme reduces the NADPH available concentration, which leads to a reduction of PBC antioxidant mechanisms [97].

In addition to this, there is a decrease in expression and activity of key transcription factors such as PDX1 and MafA. This affects the regulation of multiple genes implied in the function of PBC, including proinsulin [98]. Likewise, PBCs are especially sensitive to ER stress due to their high rates of proinsulin biosynthesis in response to glucose stimulation. Glucotoxicity causes an increase in insulin synthesis, and this leads to the accumulation of unfolded proteins in the interior of the ER, conducting to the activation of a defense mechanism executed by the PBC known as unfolded protein response (UPR). However, its exaggerated activation can induce molecular mechanisms that lead to cell death [99].

## 9. Lipotoxicity

At the cellular level, FFA are generated through de novo synthesis and/or degradation of triglycerides and phospholipids by cellular lipases. They can also be imported to the cell through transporter proteins known as lipoproteins; this can occur when there is high demand or when the extracellular concentration of FFA is high. FFA derived from each of these processes can be used for biosynthesis of cell membranes, energy production through *β*-oxidation, generation of signaling molecules of a lipid nature, posttranslational modification of proteins, and transcription regulation [100].

A number of studies have demonstrated that FFA may induce PBC death through apoptosis in the presence of high glucose concentration [101]. Acute FFA overload in the PBC amplifies insulin secretion, but, as it happens with elevated levels of glucose for a prolonged period, it causes PBC dysfunction, including inhibition of insulin secretion, inhibition of the necessary genes for cell differentiation, and promotion of its apoptosis, a process that has been named lipotoxicity [102].

Intracellular lipid storage might mediate cytoplasmic secondary signals to fatty acid esterification, like the synthesis of triglyceride due to over expression of diacylglycerol acyltransferase 1, which produces inhibition of insulin synthesis mediated by glucose without interfering in gene expression [103]. Similarly, it generates an increase in de novo synthesis of ceramides, capable of affecting at a transcriptional level of the expression of the insulin gene through the induction of JNK activation, inhibiting transcription through a c-Jun-dependent pathway. Alternatively, ceramides inhibit protein kinase B, allowing the Foxo1 transcription factor to act at the nuclear level, repressing genes with nuclear PDX1 exclusion [104]. However, this inhibition mechanism is not exclusive to ceramides because it has been observed that palmitate can directly inhibit PDX1 and MafA [103].

Other mechanism that should be considered as lipotoxicity mediators is ER stress, caused by saturated fatty acids like palmitate in the PBC. This leads to calcium ion loss through the calcium channels of the ER. The second mechanism that should be considered is the increase in ROS concentration, a product of the beta-oxidation of the fatty acids that surpasses the antioxidant mechanisms of the PBC [105, 106].

The effect of the FFA over PBC apoptosis *in vitro* is difficult to interpret due to various reasons. There are significant differences between clone primary cells and PBC regarding their sensitivity to cytotoxic effects. Additionally, FFA concentrations used *in vitro* vary according to different researchers [107]. Furthermore, it is important to highlight that the effects FFA have over PBC will depend on the length and saturation degree of the carbonated chain [108]. In a pilot study, Busch et al. demonstrated that the level of expression of the stearoyl-CoA desaturase enzyme correlates to PBC resistance to the proapoptotic effect of palmitate. This indicates that the capacity to desaturate FFA might have a protective effect against lipotoxicity [109].

## 10. Apoptosis Mediated by Endoplasmic Reticulum Stress: Role of the Islet Amyloid Polypeptide

Currently, it is known that dysfunction, apoptosis, and loss of PBC mass play a fundamental role in the pathogenesis of DM2 and that these phenomena are precipitated by metabolic stress states such as IR [110]. Studies have demonstrated that the death of these cells is due to aggregates of amyloid at the intracellular and extracellular levels, which then form toxic oligomers capable of altering the structural and functional stability of the cellular membranes [111]. IAPP is the main component of these aggregates [112]; however, there is evidence that this polypeptide also plays an important role in apoptosis mediated by ER stress, similarly conducting to PBC death [113].

IAPP or amylin is a polypeptide pancreatic hormone formed by 37 amino acids, and it is synthetized, stored, and secreted with insulin by the PBC of the islets of Langerhans, although its synthesis has also been described in the gastric endocrine cells. Since it is cosecreted with insulin, it is to be expected that both hormones share the same stimuli for their release [114].

Its synthesis initiates as a preprohormone with 89 residues that, after being split to its preform of 67 residues in the ER, it reaches its final conformation in the secreting vesicles of the PBC due to endoproteases PC2 and PC1/3 and carboxypeptidase E (CPE) activity [115]. This neurohormone participates in the control of the gastric emptying, inhibition of glucagon release, glucose homeostasis, and satiety regulation [116].

In physiological conditions, its concentration is inferior to that of insulin; however, at the onset and development of diseases like DM2, its production rises as a consequence of compensatory hyperinsulinemia, characteristic of the IR state [114]. Eventually, the greater protein expression by the PBC leads to ER stress which implies an accumulation of unfolded proteins inside the ER, activating the UPR mechanisms [117].

Three key UPR sensors mediate the correct functioning of the ER: activating transcription factor 6 (ATF6), inositol-requiring enzyme 1 (IRE1), and PKR-like ER kinase (PERK). These will activate regulation pathways that allow for the decongestion and renormalization of the ER activity [114]. Only when these fail in their attempt at maintaining cell homeostasis will they induce pathway activation that leads to apoptosis. To this purpose, chronic stress of the ER activates IRE1, PERK, and ATF6, which then will activate CCAAT-enhancer-binding protein homologous protein (CHOP) transcription, a protein that is normally at low concentrations in the cytoplasm but that, once activated, increases its concentration and is translocated to the nucleus, where it promotes DNA fragmentation and stops the cell cycle [118].

In addition, there are other proapoptotic pathways activated as a response to ER stress. IRE1 activates the JNK pathway and the apoptosis-signal-regulating kinase 1 (ASK1) cascade. This similarly promotes cell death [119].

Conversely, one of the substrates of the PI3K/Akt pathway, glycogen synthase kinase 3 *β* (GSK*β*), also has an important role in PBC apoptotic mechanisms. ER stress attenuates the phosphorylative capacity of Akt, which leads to GSK*β* dephosphorylation and subsequent apoptosis induction [120].

**Figure 3:**
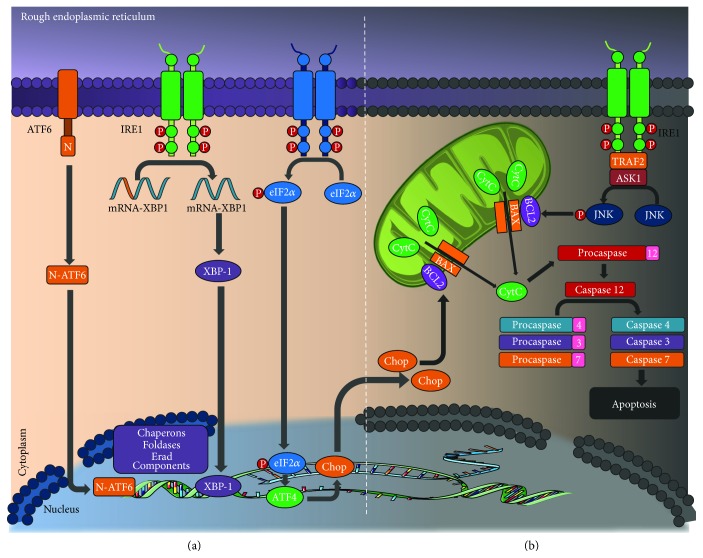
Unfolded protein response. (a) Homeostasis: activating transcription factor 6 (ATF6), inositol-requiring enzyme 1 (IRE1), and PKR-like ER kinase (PERK) mediate the correct functioning of the ER. These regulate pathways that allow for the decongestion and renormalization of the ER activity. (b) Apoptosis intrinsic pathway: chronic stress of the ER activates IRE1, PERK, and ATF6, which then will activate CCAAT enhancer-binding protein homologous protein (CHOP) transcription. This promotes DNA fragmentation and stops the cell cycle and another proapoptotic pathway (see text). ATF6: activating transcription factor 6; IRE1: inositol-requiring enzyme 1; eIF2*α*: eukaryotic initiation factor 2; mRNA: messenger ribonucleic acid; XBP-1: X-box binding protein 1; ERAD: endoplasmic reticulum-associated protein degradation; CHOP: CCAAT enhancer-binding protein homologous protein; Bcl2: B-cell lymphoma 2; BAX: Bcl-2-like protein 4; CytC: cytochrome c. TRAF2: TNF receptor-associated factor 2; ASK1: apoptosis signal-regulating kinase 1; JNK: c-Jun N-terminal kinase; ATF4: activating transcription factor 4.

Finally, the caspase pathway represents the convergence point of the majority of proapoptotic signals. Procaspase 12 is the specific mediator of apoptosis by ER stress in rats; its human equivalent is caspase 4 [121]. The signaling mechanism subsequent to caspase 12 activation is still unknown; however, studies have reported caspase 2, 8, and 9 as the initiators of the pathway and caspases 3, 4, and 7 as the executioners [119] (Figure 3).

Summarizing, when the ER of the PBC loses its homeostasis as consequence of insulin and IAPP production, the UPR mechanisms are hyperactivated. These will attempt to reestablish the function of the organelle and if they fail, they will facilitate proapoptotic pathway activation, which then will end in a reduction of PBC mass in DM2 patients.

## 11. Autophagy: Two Faces of the Same Coin

Autophagy is a type of cell death dependent on the lysosomal machinery that physiologically degrades and recycles cell components such as organelles and unfolded proteins [122]. This type of cell death is described as the accumulation of numerous lipid vesicles in the PBC cytosol. There are at least three well-described types of autophagy, among these; macroautophagy plays an important role in the survival of this cell group [123]. Macroautophagy is characterized by the reordering of membranes in order to envelop cell components—in isolation membranes—that will later be degraded [124].

The presence of diverse stressors activates autophagy, and among the most important there are oxidative stress and ER stress [125]. The central pathway starts with the formation and expansion of an isolation membrane in the shape of a cone called preautophagosome, which envelops the cytoplasmic components and forms the autophagosome that will fuse with the lysosome, rich in hydrolytic enzymes that degrade cell components [126]. This involves at least five molecular components that contain the autophagy-related proteins (ATG): the activating complex UNC-51-like kinase (ULK1)/ATG1, the Benclin/PI3K (VPS34) complex, two transmembrane proteins (ATG9 and VMPL), two ubiquitin-like conjugation systems (ATG12-ATG5 and ATG8/LC3), and five proteins that mediate the union between the phagosome and the lysosome [127].

In the presence of nutrients, autophagy is inhibited by the activation of the mTORC1 complex, a modulator activated during the insulin pathway or in states of abundant nutrients. Conversely, during inanition or intracellular energy reduction (with AMPK activation), or in the presence of mTOR inhibitors, ATC proteins can be recruited to form a complex that will initiate autophagy [128].

Autophagy is divided into three fundamental processes, starting with the *activation* by dephosphorylation of the ULK complex (this contains ATG13, FIP200, and ATG101 proteins), in which mTOR dissociates provoking its activation [129]. One of its target proteins is the autophagy/beclin-1 regulator 1 (AMBRAl). After its phosphorylation, AMBRAl releases the cytoskeleton benclin-1/PI3K class III complex, allowing for autophagy initiation [130]. In the *nucleation* process, the ULK complex activates the benclin-1PI3K class III for the allosteric activation of PI3K that initiates the formation of phosphatydil-inositol-3-phosphate (PIP3). PIP3 recruits executioners like the double FYVE domain-containing protein (DFCP1), and it promotes formation of the omegasome and the recruitment of the necessary WIPI for its maturation. This complex is negatively regulated by PI3K/akt class I and by antiapoptotic signals like Bcl-2 [131].

**Figure 4:**
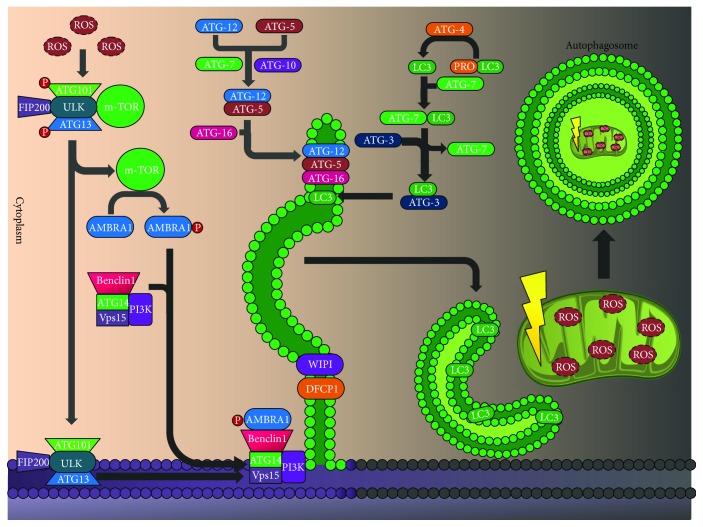
Autophagy: the presence of diverse stressors activates autophagy. It is divided into three fundamental processes: activation, nucleation, and elongation or expansion. Autophagy maintains the microarchitecture of the pancreatic beta cell (see text). ROS: reactive oxygen species; ATG101: autophagy-related 101; FIP200: FAK family kinase-interacting protein of 200 kDa; ULK: unc-51-like autophagy-activating kinase 1; ATG13: autophagy-related 13; ATG12: autophagy-related 12; ATG7: autophagy-related 7; ATG10: autophagy-related 10; ATG5: autophagy-related 5; ATG16: autophagy-related 16; ATG14: autophagy-related 14; mTOR: mammalian target of rapamycin; AMBRA1: autophagy/beclin-1 regulator 1; Vps15: serine/threonine-protein kinase VPS15; PI3K: phosphoinositide 3 kinase; LC3: microtubule-associated protein 1A/1B-light chain 3; WIPI: WD repeat domain phosphoinositide-interacting protein; DFCP1: double FYVE domain-containing protein.

Lastly, there is the *elongation or expansion* process that is initiated with the covalent conjugation of ATG12-ATG5, with the collaboration of ATG7 and ATG10, forming an association complex with ATG16 in a noncovalent union in order to form a multimeric ATG12-ATG5-ATG10 complex that acts as the E3 ligase for LC3. Finally, conjugation of phosphatidylethanolamine to an LC3 glycine residue causes the sequence activation of proteases ATG4, ATG7 (E1-like enzyme 1), and ATG3 (E2-like enzyme 2), producing the transformation of LC3 from its soluble form (LC3i) to the form associated with the autophagy vesicle (LC3ii). This LC3 lipid form leads to the formation of the double membrane that engulfs the intracellular content, and it forms the autophagosome that then will fuse with lysosomes where the sequestered material will be degraded by hydrolytic enzymes [132] (Figure 4).

The fundamental physiological role of autophagy is reorganization of nutrients, from unnecessary processes to the most important for the survival of the cell. In the PBC, autophagy maintains the cell microarchitecture [133]. Therefore, it is necessary for the maintenance of an adequate insulin granule pool, controlling their degradation [134]. In addition, it is important in inanition periods where it controls insulin secretion by the PBC in order to maintain euglycemia, inhibiting autophagy and selectively degrading new secretory granule through lysosomes in order to maintain low insulin secretion during fasting periods [135].

When IR is present, there is a rise in FFA influx that can act as an activator and potentiator of autophagy of the PBC through JNK1 activation, independent from oxidative and ER stress [136]. This induction mainly takes place with long-chained fatty acids, such as palmitate [137]. However, when there is chronic exposure to these fatty acids, there is a disruption of autophagy characterized by the lysosomal reduction with intracellular toxic effects [138].

Actually, in *in vitro* and *in vivo* models with deletion of the *atg7* gene, a greater expression of mitochondrial dysfunction markers [139] and ER stress due to the decrease of expression of the genes related with the UPR machinery can be observed. This leads to the progressive reduction of pancreatic mass and a lesser glucose tolerance [133]. These effects seem exaggerated by stressors like obesity [140], which predisposes for DM and complications like diabetic nephropathy. Furthermore, autophagy controls toxic intracellular accumulation, but in an environment in which one of these (e.g., pancreatic islet polypeptide in its monomeric form) rises in an exaggerated manner, inhibiting autophagy and making the PBC more susceptible to toxicity dependent on said toxic component [141].

Early stages of DM2 are characterized by a PBC adaptive response to IR in which cellular hyperplasia is produced, there is ATP production increase, and insulin synthesis rises. In this context, this type of death is necessary for PBC survival once the mitochondrial and unfolded protein degradation mechanisms that could initiate the apoptotic pathway are in place, permitting PBC compensatory hyperplasia [142, 143]. Therefore, intrinsically activated autophagy at low levels is necessary at the pancreatic level, and any dysfunction in this process might predispose or worsen the prognosis for DM [144].

Conversely, in certain conditions, the degradation of cellular components could induce PBC death, although this has not been completely described. In individuals with DM2, a greater autophagy activity has been observed (PBC death with excessive vacuole accumulation without chromatin condensation), which could contribute to pancreatic mass loss [125]. In an *in vitro* and *in vivo* model of insulinoma cells performed in rats (MIN6) with deficiency of the pancreatic and duodenal homeobox 1 (PDX1), the presence of autophagy was found before the beginning of apoptosis, and once inhibited, there was greater PBC survival [145]. Future studies should investigate the true role of autophagy, considering the potential therapeutic implications it could have.

### 11.1. Ubiquitin-Proteosome System and Pancreatic Beta Cell Dysfunction

The ubiquitin-proteosome is a heavily controlled, ATP-dependent intracellular proteolysis system, with the purpose of degrading unnecessary (or damaged) proteins as part of the unfolded protein response (UPR), regulating cell cycle, cell death, and overall transduction processes [146]. PBC requires a highly efficient UPR system, basically due to the highly protein synthesis demand of the cell to maintain homeostasis. During oxidative stress or ER stress, autophagia and ubiquitin-proteosome system prevent accumulation and aggregation of excess proteins, which could lead to toxicity [147].

In patients with DM2, there is increased proteasome activity in nucleus and cytoplasmic compartments, which seem to lower in those patients who are treated with insulin, which is given as replacement therapy and, ergo, reduces PBC stress levels derived from prolonged insulin secretion activity [148]. One of the factors associated with dysfunctional proteasome activity is mediated by the accumulation of IAPP, which lowers the expression of UCH-L1 protein (ubiquitin hydrolase), leading to ER stress and increased levels of cleaved caspase 3, activating apoptosis [148].

During chronic hyperglycemia, the proteolytic activity of trypsin, caspases, and chymotrypsin is affected *in vivo*, associated with increased ubiquinated proteins, ER stress, and caspase 3-mediated apoptosis [149]. These effects are ameliorated with the use of Exadin-4, a GLP-1 analog, which prevents proteasome dysfunction and increases PBC survival in hyperglycemic conditions [150]. Likewise, lipotoxicity mediated by palmitate lowers proteasome activity, inducing ER stress and it lowers the expression of prosurvival proteins, such as Bcl-2 and Bcl-XL [151].

## 12. Pyroptosis: Another Oxidative Stress Execution Pathway

Even though proinflammatory cytokine activity has been widely studied in DM1, its activity in the appearance and development of DM2 seems significant, even for a therapeutic purpose [152]. Pyroptosis is a type of programmed cell death morphologically and mechanically different to other types of cell death. It is characterized for being proinflammatory and mediated by IL-1*β*. The death execution mechanism is managed through caspase 1 activation (not related with apoptotic cell death) and the formation of a macromolecular complex known as inflammasome [152, 153]. Activation of caspase 1 leads to the splitting and activation of IL-1*β*, a proinflammatory cytokine related to IR and PBC function [154, 155], and it causes edema, the formation of a pore, and cellular lysis, unlike apoptosis.

The inflammasome is a protein complex that acts as a sensor for pathogen-associated molecular patterns (PAMPs) or damage-associated molecular patterns (DAMPs) [156]. The NLR3P inflammasome seems like an important interaction pathway between pyroptosis, mitochondrial damage, and autophagy in DM2. It is activated by ROS generation by the mitochondria, mitochondrial DNA release, or cardiolipin and cathepsins release to the cytosol after lysosomal destabilization [157, 158].

Similarly, inflammasome components participate in cell response; they are capable of activating autophagy at its earliest stages (with the purpose of degrading cytosolic components) or activating pyroptosis with chemotactic purposes [153]. Autophagy seems to protect cells from pyroptosis degrading mitochondria and inflammasome components, thus limiting ROS production [159]. Conversely, when the NLR-procaspase 1 complex is activated, formed, and interacted with beclin-1/Atg6, it inhibits autophagy and promotes pyroptosis [160].

Likewise, inflammasome could participate in the inflammatory response mediated by macrophage in the adipocyte, inducing obesity and IR [161, 162]. An increase of IL-1*β* expression as a response to chronic hyperglycemia has been observed in PBC [163]. However, the common mechanism of all of these events is the generation of great ROS quantities, which provokes separation of a heterodimeric complex with sensor function constituted by TXNIP-TRX, where TRX induces NLRP3 inflammasome assembly and the activation of procaspase 1 [164]. To this sense, caspase 1 can induce cell death through the following: (a) micropore formation in the plasmatic membrane that allows the entrance of water or ion exchange; (b) IL-1*β* activation that would act in an autocrine manner and will generate cell death through the previously described mechanisms [154].

**Figure 5:**
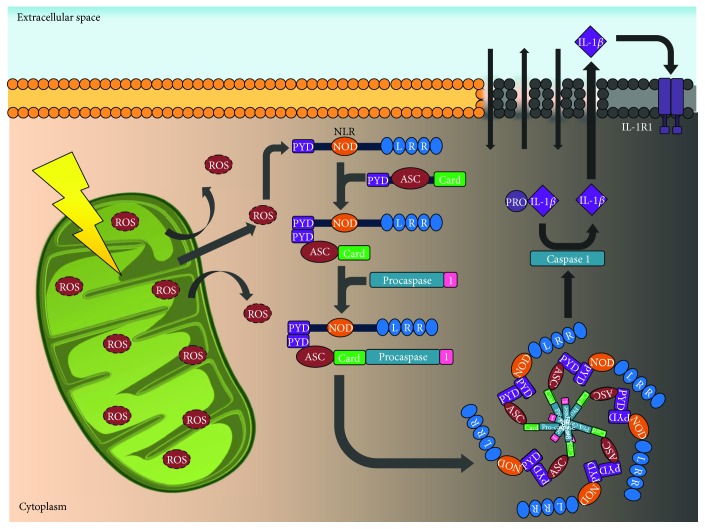
Pyroptosis: IL-1*β* causes activation of caspase 1 and the later formation of the macromolecular complex like the inflammasome, which interacts with other mechanisms that have been previously described. This favors ROS production at the mitochondrial level, mitochondrial DNA release, or cardiolipin and cathepsins release to the cytosol after lysosomal destabilization (see text). ROS: reactive oxygen species; PYD: N-terminal pyrin domain; NLR: nod-like receptors; NOD: nucleotide-binding oligomerization domain protein; ASC: apoptosis-associated speck-like protein containing CARD; CARD: caspase recruitment domain; IL-1*β*: interleukin 1 beta; IL-1R1: interleukin 1 receptor type 1.

Although these mechanisms have not been reproduced in all experimental models, evidence seems to show that oligomerization of human IAPP is fundamental for cell death induction dependent of inflammasome once it activates NLRP3 [165]. However, the main IL-1 producer in response to IAPP oligomerization is not PBC, but the residing macrophages infiltrate the pancreatic islets [166], altering mediator proinflammatory/anti-inflammatory intraislet balance to a proinflammatory environment. In the PBC microenvironment, chronic exposition to IL-1*β* conditions for oxidative stress through the expression of nitric oxide synthase (NOS) stimulates ROS production [167]. Similarly, one of the target transcription factors for this cytokine is the activation of NF-*κ*B, which has been involved in the increase of NOS and IL-1*β* autostimulation, and it promotes PBC dysfunction [163, 168] (Figure 5).

Finally, pyroptosis seems to mediate target organ damage in a greater measure, and the myocardium is one of the most analyzed tissues in experimental models. As it occurs in PBC, chronic hyperglycemia appears to increase ROS production on this tissue, which then promotes NF-*κ*B translocation to the nucleus and the expression of TXNIP, regulating the activity of the inflammasome and its caspase 1-dependent executioner mechanisms [169]. One of the most relevant effects is observed in cardiac remodeling, with damage to the ultrastructure and myocardiocytes. This comprises myofibril destruction, mitochondrial edema with disorganized crests, glycogen excess lysis, lipid accumulation, and interstitial fibrosis (increase of the collagen I/collagen III ratio) [169]. Regression of these effects with the NLRP3 blockage through pharmaceutic intervention and/or microRNAs must be evaluated in the upcoming years, with the purpose of understanding the true reach of this therapy in the clinical setting [170, 171].

## 13. Ultrastructural Changes during Pancreatic Beta Cell Death

The processes associated with DM development modify the ultrastructure of PBCs, which can be observed via electronic microscopy and involve several organelles [172]. The changes seem to be specific to type of cell death and can be used to determine which diabetes type has been developed [173].

In subjects with DM1, PBCs do not show changes in number and density of insulin granules and mitochondria are preserved in number. However, they acquire a tubular/elongated shape, ERs are more visible and numerous, and the attached ribosomes are more electrodense. Finally, autophagic vacuoles are hardly observed [174, 175]. In regard to cell death mechanism, PBCs showed signs of apoptosis: citoplasmatic condensation, nuclear fragmentation, preserved plasma membrane, and formation of apoptotic bodies [172, 176, 177].

In regard to DM2 patients, ER cisterns are more centralized, with numerous electrodense ribosomes. Mitochondria exhibit multiple shapes resulting from increased microchondrial dynamics (fission and fusion), which eventually lead to mitochondrial loss; mitochondria near the ER are usually round and bloated [175]. In regard to insulin granules, they are decreased in number and density, associated with dilated ER cisterns [178–180]. Among the type of PBC deaths, apoptosis and autophagy have been greatly studied. These PBCs show signs of autophagy vacuoles, including a double membrane with subcellular structures sequestered in them, such as mitochondria, insulin granules, and others, without any evident nuclear alteration [175, 181–184]. Cells that die via pyroptosis also have distinguishable features [185], such as increased size and DNA fragmentation, which is like apoptosis but without mitochondrial permeation [186].

### 13.1. Metabolic Interactions during PBC Death in DM1 and DM2

It has been suggested in the past years that PBCs may share similar cell deaths during DM1 and DM2. It has been reported that glucotoxicity and lipotoxicity affect proinflammatory cytokine profiles. PBCs exposed to hyperglycemia *in vitro* generate increased expression of IL-1 *β*, activating NF-*κ*B pathway and FAS signaling, which culminates in apoptosis [187]. Likewise, free fatty acids can also activate the NF-*κ*B pathway, suggesting that the common pathway in diabetes development is the IL-1*β*/NF-*κ*B pathway [188]. Palmitate is also associated with de novo synthesis of ceramide, which phosphorylates p38 and JNK [189], activating inflammation and cell death [190–192].

## 14. Conclusion

DM is a chronic disease that annually affects a growing number of people worldwide. Therefore, identification of the diverse etiopathogenic phenomena implicated in its appearance is a subject of ample investigation in current diabetology. Consequently, PBC progressive dysfunction is not only limited to a simple apoptotic process, as it occurs during the “immune-mediated attack” in the natural history of DM1, but it also involves diverse alternative pathways that involve different cytokines, caspases, and intracellular mediators capable of generating PBC death in this group of patients. Likewise, diverse mechanisms that occur parallel to glucotoxicity and lipotoxicity processes have been found in DM2 patients. These processes involve IAPP and UPR, as well as inflammasome formation or the loss of regulation of physiological autophagy. The entirety of these new mechanisms represents the meeting point of immunometabolism, a new and promising component of what once was known as the ominous “octet” of DM2.
